# Clinical performance of BioCoat™ and Fissurit FX: a randomized 18-month split-mouth study

**DOI:** 10.1007/s10266-026-01332-4

**Published:** 2026-02-02

**Authors:** Esra Ergün, Canan Bayraktar Nahir

**Affiliations:** Department of Pediatric Dentistry, Faculty of Dentistry, Tokat Gaziosmanpaşa University, Tokat, Türkiye

**Keywords:** Pit and fissure sealant, BioCoat™, Split-mouth design, Bioactive resin, Clinical performance

## Abstract

Pit and fissure sealants (PFS) are effective in preventing occlusal caries. This randomized split-mouth clinical study aimed to compare the 18-month clinical performance of the bioactive resin-based PFS (BioCoat™) with a fluoride-containing conventional resin-based PFS (Fissurit FX) and to assess the influence of patient-related factors on material success. Sixty-three pediatric patients (aged 7–9) requiring non-invasive PFS were included. BioCoat™ (Group 1) and Fissurit FX (Group 2) were applied to contralateral mandibular permanent first molars. Clinical evaluations were performed at 3rd, 6th, 9th, 12th, and 18th months using modified USPHS criteria. Kaplan–Meier survival analysis and logistic regression were applied. At 18 months, success rates were 90.2% for BioCoat™ and 88.2% for Fissurit FX, with no statistically significant difference between groups (*p* > 0.05). Both materials showed similar performance in marginal discoloration–adaptation, retention and secondary caries (*p* > 0.05). Median survival times were 17.72 months (BioCoat™) and 17.49 months (Fissurit FX), showing no significant difference (*p* = 0.744). No significant effect of patient-related factors on material success was determined (*p* > 0.05). BioCoat™ demonstrated comparable long-term clinical performance to Fissurit FX. Its bioactive properties may provide additional preventive benefits, supporting its safe use in pediatric dentistry.

## Introduction

Dental caries is a major public health problem that is frequently seen in childhood and is characterized by mineral loss caused by microbial biofilm [1]. According to the World Health Organization, dental caries affects approximately 60–90% of school-aged children. This prevalence highlights the need for preventive approaches based on early intervention [2]. Deep fissures, particularly those located on occlusal surfaces, are highly susceptible to caries development due to their complex morphology, which hinders effective mechanical cleaning. Pit and fissure sealants (PFS) help isolate these anatomically vulnerable sites, thereby preventing bacterial plaque accumulation and potentially arresting the progression of dental caries [3]. The American Dental Association (ADA) and the American Academy of Pediatric Dentistry (AAPD) recommend the early application of PFS in individuals at elevated caries risk [4]. Due to their ease of application and long-term effects, PFS are widely preferred in preventive pediatric dentistry [5].

Conventional resin-based PFS provide high retention; however, their technique sensitivity to moisture control and the risk of microleakage due to polymerisation shrinkage remain notable limitations [6]. Glass ionomer-based materials, which are preferred for their fluoride release and moisture tolerance, have limitations in terms of retention [7]. In response to these limitations, bioactive resin-based PFS have been developed that demonstrate both protective efficacy and material-specific advantages [8].

Bioactive PFS not only acts as a physical barrier, but also exhibits a biological effect by supporting remineralization [9]. In this context, BioCoat™ (Premier, USA) has been developed with SmartCap™ technology, which prevents demineralization and inhibits the progression of cavities by providing controlled release of fluoride and calcium ions. In addition, its high filler content, low polymerization shrinkage, and improved wear resistance contribute to its favourable physical properties [10].

Although several in vitro studies have evaluated the multifaceted properties of BioCoat™, including shear bond strength, remineralization potential, micromechanical adhesion, and flexural strength [8, 10–15], the number of clinical studies remains extremely limited. To date, only one study has qualified as a clinical trial with a 12-month follow-up period [16]. In the current literature, there is insufficient scientific evidence regarding the long-term clinical efficacy of BioCoat™. This deficiency clearly highlights the need for high-quality clinical studies that will objectively demonstrate the material's long-term performance.

The present study was designed to provide a novel contribution to the literature by comparing the clinical performance of a bioactive resin-based PFS (BioCoat™) with a conventional fluoride-containing resin-based PFS (Fissurit FX) over an 18-month period using a split-mouth design. More specifically, the primary objective was to evaluate and compare the clinical performance of BioCoat™ and Fissürit FX throughout the 18-month follow-up period. The secondary objective was to investigate the influence of individual factors on material success. Accordingly, the null hypothesis tested was that there would be no statistically significant difference in clinical success between the BioCoat™ and Fissürit FX at the end of the 18-month follow-up period.

## Materials and methods

### Study design

This prospective, randomized clinical trial was designed with a split-mouth design. This study was approved by the Clinical Research Ethics Committee of Tokat Gaziosmanpaşa University Faculty of Medicine (Approval No: 23-KAEK-150, dated 22 June 2023). The study protocol was registered in the clinical trials database (www.clinicaltrials.gov) under the identifier NCT06987955. The study was conducted in accordance with the principles of the Declaration of Helsinki and reported in compliance with the Consolidated Standards of Reporting Trials (CONSORT) guidelines. In compliance with ethical standards, written informed consent was obtained from the parents of all participating children. Following the ethical approval for this study, between June 25, 2023, and August 31, 2023, pediatric patients aged 7–9 years who presented for routine examination and treatment at the Department of Pediatric Dentistry, Faculty of Dentistry, Tokat Gaziosmanpaşa University, were included in the study. All eligible participants received clinical interventions (non-invasive PFS application on their permanent mandibular first molars) during this period. Each patient was then followed up for 18 months, with the final follow-up completed on March 1, 2025.

### Sample size calculation

The required sample size for this study was calculated using GPower software version 3.1.9.2 (Franz Faul, University of Kiel, Germany). Based on a 95% confidence level (1 − α), 95% statistical power (1 − β), and an effect size of w = 0.369 derived from previously published split-mouth clinical trials with similar outcome measures, the minimum required sample size was calculated using a “Goodness-of-fit tests: Contingency tables” at the tooth level. Accordingly, a total of 114 teeth (57 patients) were required [17]. To account for potential loss to follow-up, this number was increased by 10%, resulting in a final planned sample size of 126 teeth (63 patients).

### Study population

Inclusion criteria for the study were as follows: written informed consent obtained from both the patient and their parent or guardian; agreement to attend scheduled follow-up visits; absence of acute or chronic systemic disease; no physical or mental disability; no history of allergy to any medication or dental restorative material; absence of parafunctional habits, such as bruxism or clenching; no occlusal disturbances due to orthodontic anomalies; and a cooperative behaviour rating of 3 or 4 according to the Frankl Behaviour Rating Scale.

Tooth-level inclusion criteria were defined as follows: complete eruption of the occlusal surface of the permanent mandibular first molar; presence of symmetrical contralateral teeth; and a clinical indication for non-invasive PFS application on these molars. In addition, the mandibular first molars selected for PFS application were required to be adjacent to primary second molars, and the opposing arch had to contain both permanent first molars and adjacent primary second molars.

Exclusion criteria included children with a cooperation level of 1 or 2 according to the Frankl Behavior Rating Scale; previously restored permanent first molars; presence of fluorosis, molar-incisor hypomineralization (MIH), dysplasia, or any hypomineralized areas on the target teeth; and children exhibiting unilateral chewing habits or a tendency to retain food in the oral cavity.

### Participant and ındividual characteristics assessed at baseline

Demographic and clinical data of the participants were recorded including age, gender, dfs score [d: number of decayed surfaces, f: number of filled surfaces, and s: total surfaces], gingival index (GI), plaque index (PI), and individual caries risk classification.

To assess the eligibility of the teeth to be included in the study, caries-free status was evaluated using the DIAGNOdent pen device (KaVo, Germany). Teeth presenting a DIAGNOdent reading of ≤ 24 were considered clinically sound and included in the study.

### Blinding

The study was conducted using a single-blind design. Patient blinding was achieved, because participants did not know which material was applied to which tooth. However, due to the distinct color differences between the materials, operator blinding was not possible, because the operator could identify the material during application.

### Randomization

The permanent mandibular first molars (teeth 36 and 46) included in the study were evaluated using a split-mouth design. In each patient, two different PFS were applied to these molars: BioCoat™ (Group 1) and Fissurit FX (Group 2). The distribution of the PFS material to each tooth was determined using a computer-assisted randomization program (https://www.random.org/). Suitable teeth were numbered as 1 and 2, starting from the same quadrant in each patient, and assigned randomly according to these numbers. The randomization sequence was generated by an independent researcher who was not involved in the clinical procedures or outcome assessment. Allocation concealment was ensured by keeping the randomization results inaccessible to the operator until the time of material application.

### Clinical practice protocol

To ensure operator consistency, all PFS applications were performed by a single clinician (E.E). Isolation of the included teeth was achieved by applying rubber dam. In accordance with the manufacturers’ instructions, a 37% phosphoric acid gel was applied to all PFS on the occlusal surfaces of the target teeth and left in place for 20 s. The acid was then rinsed off with a water spray for 20 s and the teeth were dried with an air spray for 10 s.

Group 1: BioCoat™ PFS was applied to one of the mandibular permanent first molars.

Group 2: The mandibular permanent first molar on the opposite side was applied with Fissurit FX.

Both PFS materials were evenly distributed throughout the all PFS using a clean dental probe to ensure homogenous coating and prevent air bubble formation. Polymerization was performed for 20 s using a Valo Cordless curing light device (Ultradent, USA). Following polymerisation, residual monomers were gently removed by wiping the tooth surfaces with a moist cotton pellet. Occlusal contacts were then assessed using articulating paper, and adjustments and polishing were performed using finishing burs as needed.

### Follow-up

All PFS were evaluated by the same clinician (E.E.) using modified United States Public Health Service (USPHS) criteria (Supplementary Material). Evaluations were performed at 3, 6, 9, 12, and 18 months, focusing on marginal discoloration, marginal adaptation, retention, and the presence of secondary caries. According to the modified USPHS criteria, PFSs that scored “Alpha” or “Bravo” in all parameters were considered clinically successful. In contrast, any PFS receiving a “Charlie” score in any category was considered unsuccessful, replaced using the conventional technique (acid etching, rinsing, and application of a resin-based PFS), and excluded from further analysis. To assess intra-examiner reliability, a randomly selected group of 10 patients (20 teeth) were re-evaluated after a 2-week interval under identical clinical conditions.

### Statistical analyses

Statistical analyses were performed using IBM SPSS Statistics v23 (Statistical Package for the Social Sciences, Chicago, IL, 2015). Descriptive statistics for quantitative variables were presented as mean ± standard deviation and median (minimum–maximum), while categorical variables were summarised as frequency (percentage).

Intra-examiner reliability for USPHS scoring was assessed using Cohen’s kappa statistics. Normality of quantitative data was assessed using the Kolmogorov–Smirnov test. For comparisons between two independent groups, the Independent Samples *t* test was used for normally distributed data, and the Mann–Whitney *U* test was applied for non-normally distributed data. Relationships between categorical variables were analysed using Fisher’s exact test.

Intra-group comparisons of overall clinical success rates across follow-up periods were performed using Cochran’s *Q* test, with Bonferroni correction applied for multiple comparisons. To evaluate the functional longevity of the PFS in the oral environment, Kaplan–Meier survival analysis and the log-rank test were employed. The influence of independent variables (gender, age, dfs score, gingival index, and plaque index) on the overall success of the materials was assessed using binary logistic regression analysis. A *p* value of < 0.05 was considered statistically significant for all analyses.

Due to loss to follow-up, missing data were handled using a complete-case analysis. Only participants who completed the full follow-up period were included in the final statistical analyses. An intention-to-treat analysis was considered; however, it was not feasible due to the absence of outcome data for participants lost to follow-up.

## Results

**Fig. 1:**
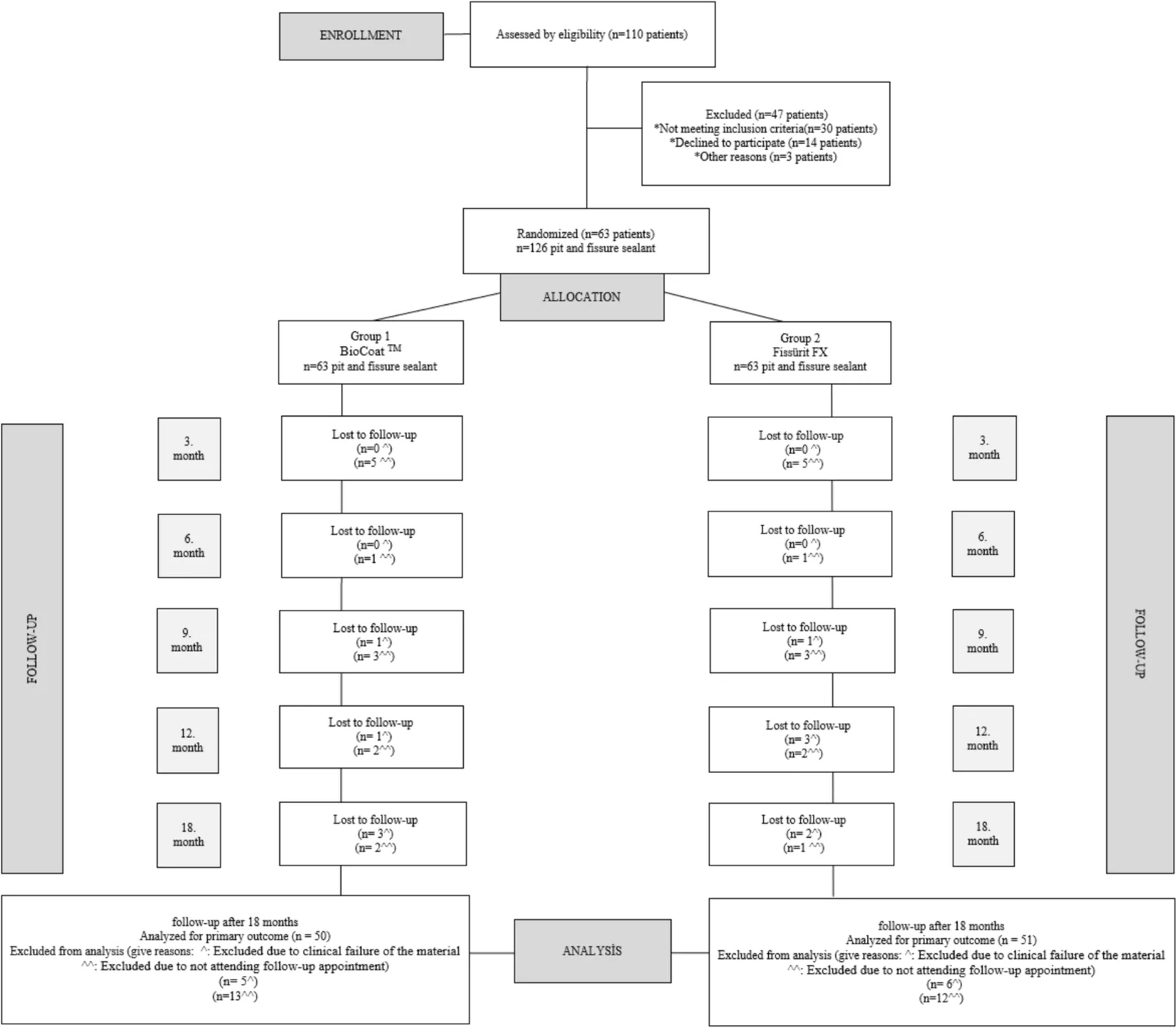
Flow chart of participants in the study. ^Excluded due to clinical failure of the material (Teeth with clinical failure (Charlie score) were excluded from subsequent follow-up evaluations). ^^Excluded due to not attending follow-up appointment

A total of 110 patients were initially assessed for eligibility. Of these, 63 patients met the inclusion criteria and received PFS applications on their mandibular permanent first molars, resulting in 126 treated teeth. The study commenced with 63 patients (126 teeth); however, 13 patients (26 teeth) were excluded due to not attending follow-up appointments (Fig. 1). Baseline characteristics of patients who completed the follow-up and those lost to follow-up were compared, and no statistically significant differences were observed between the two groups in terms of age, sex, or baseline clinical characteristics (p > 0.05). Consequently, statistical analyses were conducted on data from 50 patients (100 teeth). A post-hoc power analysis indicated that the study was completed with 92% statistical power. Intra-examiner reliability for USPHS scoring demonstrated substantial to almost perfect agreement (Cohen’s kappa = 0.86).

A total of 63 children (35 girls [55.6%], 28 boys [44.4%]) participated in the study. The mean age was 7.9 ± 0.9 years. The dfs score was significantly higher in boys (8.5 [0.00–30.00]) compared to girls (5 [0.00–19.00]) (*p* = 0.003). However, no statistically significant gender differences were observed in GI values (0.6 for both groups) (*p* = 0.844) and PI values (1.23 ± 0.62 in girls; 1.15 ± 0.49 in boys) (*p* = 0.591). Similarly, the DIAGNOdent pen scores did not differ significantly between groups, with a mean of 16.41 ± 4.84 in Group 1 and 16.41 ± 5.60 in Group 2 (*p* = 0.853). Regarding caries risk classification, 63.5% of participants were categorised as high risk, 31.7% as moderate risk, and 4.8% as low risk, with a statistically significant difference between groups (*p* < 0.001). No significant difference was found between groups in DIAGNOdent pen measurements (*p* = 0.853) (Table 1).

**Table 1:** Individual characteristics of participants assessed at the baseline

	Girl	Boy	Total	Test stat	*p* value
n (%)	35 (55.60)	28 (44.40)	63 (100.00)		
Age (years)	7.88 (6.55–9.49)	7.34 (6.55–9.53)	7.82 (6.55–9.53)	639.00	0.040*
dfs score	5 (0.00–19.00)	8.50 (0.00–30.00)	6 (0.00–30.00)	277.50	0.003^x^^,^*
GI	0.60 (0.00–1.60)	0.60 (0.00–1.50)	0.60 (0.00–1.60)	504.50	0.844^x^
PI	1.23 ± 0.62	1.15 ± 0.49	1.19 ± 0.56	0.50	0.591^y^

DIAGNOdent pen score	Group 1	Group 2	Test stat	*p* value
mean ± sd	16.41 ± 4.84	16.41 ± 5.60	1946.50	0.853^x^
median (min–max)	16 (5.00–24.00)	17 (6.00–24.00)		

Caries risk group	Low risk	Medium risk	High risk	Total	Test stat	*p* value
Girl	2 (66.70)	15 (75.00)	18 (45.00)	35 (55.60)	5.05	0.063^z^
Boy	1 (33.30)	5 (25.00)3	22 (55.00)	28 (44.40)		
Total	3 (4.80)^a^	20 (31.70)^ab^	40 (63.50)^b^	63 (100.00)		< 0.001^z^,^*^

Asterisk (*) indicates statistically significant differences (*p* 0.05)

n (%): number of children (percentage value); sd: standard deviation; min: minimum; max: maximum; test stat: test statistics; Gingival Index: GI; Plaque index: PI

^a–b^Groups labeled with the same letter are not significantly different

^x^Mann–Whitney *U* test

^y^ındependent samples *T* test

^z^Fisher’s exact test with Monte Carlo correction

The clinical performance of PFS was evaluated at 3, 6, 9, 12, and 18 months using modified USPHS criteria, based on four parameters: marginal discoloration, marginal adaptation, retention and presence of secondary caries (Table 2). The number of evaluated teeth varied across follow-up visits, because teeth that exhibited clinical failure (Charlie score) at earlier timepoints and teeth belonging to patients lost to follow-up were excluded from subsequent evaluations. In each follow-up period, the alpha, bravo, and charli score distributions for these parameters were compared between Group 1 and Group 2, and no statistically significant difference was observed (*p* > 0.05).

**Table 2:** Comparison of modified USPHS criteria between groups

Modified USPHS criteria	Score	3 months		6 months		9 months		12 months		18 months	
		Group 1	Group 2	Group 1	Group 2	Group 1	Group 2	Group 1	Group 2	Group 1	Group 2
Marginal discoloration	Alpha	58 (100.00)	58 (100.00)	57 (100.00)	57 (100.00)	54 (100.00)	53 (98.10)	50 (98.00)	48 (94.10)	45 (93.8)	42 (89.4)
	Bravo	0	0	0	0	0	1 (1.90)	1 (2.00)	3 (5.90)	3 (6.20)	5 (10.60)
	Charlie	0	0	0	0	0	0	0	0	0	0
	Total	58 (100.00)	58 (100.00)	57 (100.00)	57 (100.00)	54 (100.00)	54 (100.00)	51 (100.00)	51 (100.00)	48 (100.00)	47 (100.00)
	*p* value	-		-		1.000^x^		0.617^x^		0.486^x^	
Marginal adaptation	Alpha	57 (98.30)	58 (100.00)	52 (91.20)	55 (96.50)	45 (83.30)	46 (85.20)	42 (82.40)	42 (82.40)	37 (77.10)	37 (78.70)
	Bravo	1 (1.70)	0	5 (8.80)	2 (3.50)	8 (14.80)	8 (14.80)	9 (17.60)	9 (17.60)	11 (22.90)	10 (21.30)
	Charlie	0	0	0	0	1 (1.90)	0	0	0	0	0
	Total	58 (100.00)	58 (100.00)	57 (100.00)	57 (100.00)	54 (100.00)	54 (100.00)	51 (100.00)	51 (100.00)	48 (100.00)	47 (100.00)
	*p* value	1.000^x^		0.438^x^		1.000^y^		1.000^y^		1.000^y^	
Retention	Alpha	57 (98.30)	58 (100.00)	52 (91.20)	54 (94.70)	50 (92.60)	46 (85.20)	39 (76.50)	39 (76.50)	35 (72.90)	37 (78.70)
	Bravo	1 (1.70)	0	5 (8.80)	3 (5.30)	3 (5.60)	7 (13.00)	11 (21.50)	11 (21.50)	12 (25.00)	8 (17.00)
	Charlie	0	0	0	0	1 (1.80)	1 (1.80)	1 (2.00)	1 (2.00)	1 (2.10)	2 (4.30)
	Total	58 (100.00)	58 (100.00)	57 (100.00)	57 (100.00)	54 (100.00)	54 (100.00)	51 (100.00)	51 (100.00)	48 (100.00)	47 (100.00)
	*p* value	1.000^x^		1.000^x^		0.541^y^		1.000^y^		0.969^y^	
Seconder caries	Alpha	58 (100.00)	58 (100.00)	57 (100.00)	57 (100.00)	54 (100.00)	54 (100.00)	50 (98.00)	48 (94.10)	47 (97.90)	46 (97.90)
	Charlie	0	0	0	0	0	0	1 (2.00)	3 (5.90)	1 (2.10)	1 (2.10)
	Total	58 (100.00)	58 (100.00)	57 (100.00)	57 (100.00)	54 (100.00)	54 (100.00)	51 (100.00)	51 (100.00)	48 (100.00)	47 (100.00)
	*p* value	-		-		-		0.617^x^		1.000^x^	

n (%): number of tooth (percentage value)

Group 1: BioCoat™; Group 2: Fissurit FX

^x^Fisher’s exact test

^y^Fisher’s exact test with Monte Carlo correction

In Groups 1 and 2, demonstrated 100% clinical success at the 3rd and 6th month evaluations. At the 9th months, the success rate was 98.0% in both groups (*p* = 1.000). At 12th months, Group 1 showed a success rate of 96.1%, while Group 2 showed a success rate of 92.2%, and this difference was not statistically significant (*p* = 0.678). At 18 months, success rates were 90.2% for Group 1 and 88.2% for Group 2, with no significant difference between groups (*p* = 1.000). When comparing the intra-group follow-up periods in both groups, a statistically significant decrease in clinical success rates was observed at 18th months compared to the 3rd and 6th month clinical evaluations (Group 1: *p* = 0.006; Group 2: *p* = 0.001) (Table 3).

**Table 3:** Intergroup and ıntragroup comparison of overall clinical success rates during follow-up periods

Groups	Follow-up					Test stat	*p* value
	3 months	6 months	9 months	12 months	18 months		
Group 1	51 (100.00)^a^	51 (100.00)^a^	50 (98.00)^ab^	49 (96.10)^ab^	46 (90.20)^b^	14.333	0.006^z,*^
Group 2	51 (100.00)^a^	51 (100.00)^a^	50 (98.00)^ab^	47 (92.20)^ab^	45 (88.20)^b^	18.000	0.001^z,*^
*p* value	-	-	1.000^x^	0.678^x^	1.000^y^		

Asterisk (*) indicates statistically significant differences (*p* 0.05)

n (%), number of tooth (percentage value); Test stat, test statistics; Group 1, BioCoat™; Group 2, Fissurit FX

^a–b^Groups labeled with the same letter are not significantly different

^x^Fisher’s exact test

^y^Yates Düzeltmeli Fisher’s exact testi

^z^Cochran’s *Q* test

**Fig. 2:**
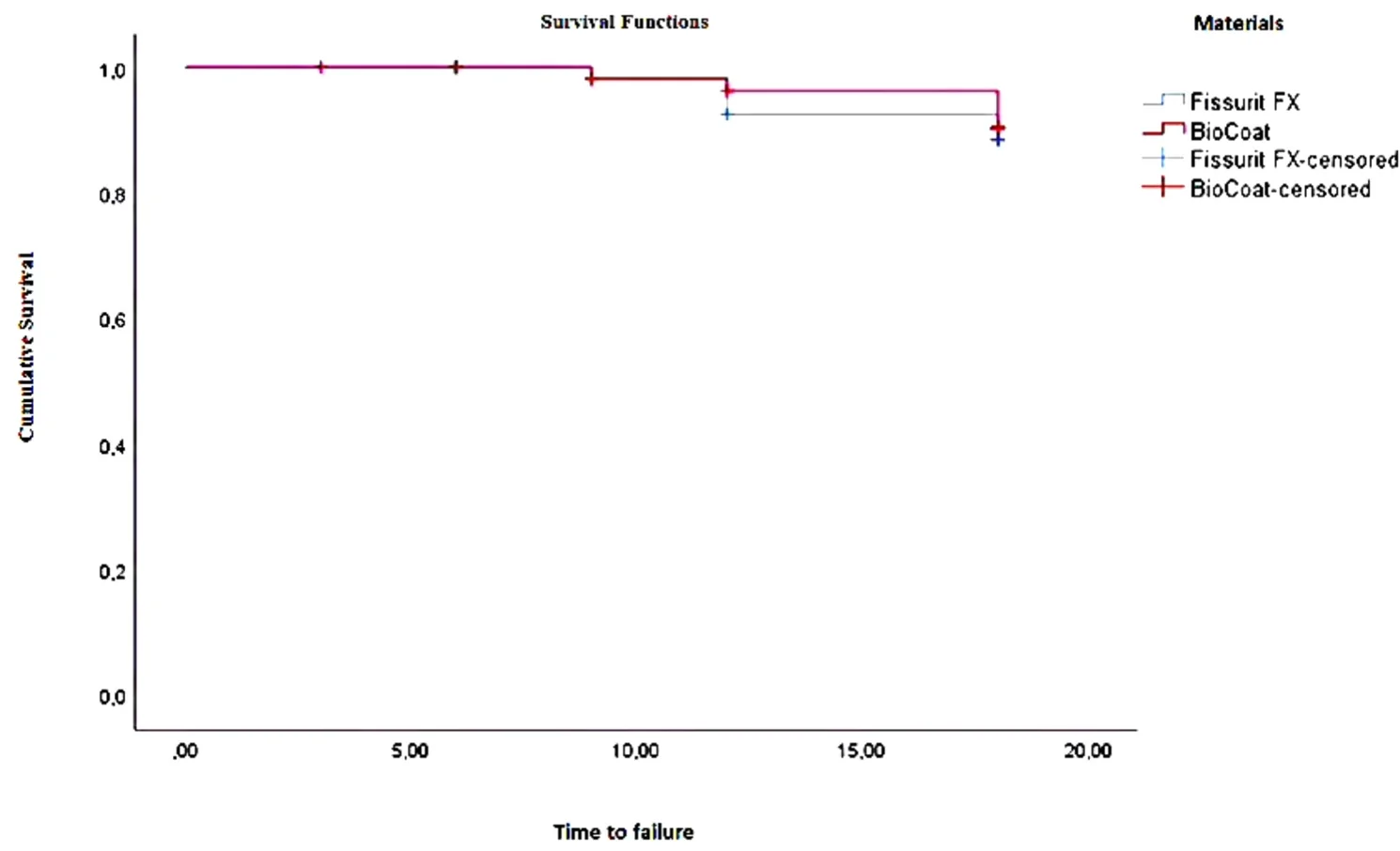
Cumulative survival probabilities of pit and fissure sealants (Fissurit FX and BioCoat™) during 18 months

According to Kaplan–Meier analysis results, the cumulative survival curves for both groups during the 18-month follow-up period are presented comparatively in Fig. 2. When the log-rank test findings were examined, the median survival time was calculated as 17.72 months (17.28–18.15) for Group 1 and 17.49 months (16.96–18.03) for Group 2 (Table 4). No statistically significant difference was found between the groups in terms of survival times (*p* = 0.744).

**Table 4:** Comparison of survival times between groups

Groups	Mean (minimum–maximum)	*p* value
Group 1	17.72 (17.28–18.15)	
Group 2	17.49 (16.96–18.03)	0.744
Total	17.61 (17.28–17.93)	

Group 1, BioCoat™; Group 2, Fissurit FX

Log-rank test

Potential factors that could affect the overall clinical success of the PFS were evaluated, including gender, age, dfs score, GI and PI. The analyses revealed that none of these variables had a statistically significant effect on the overall clinical success of teeth treated with BioCoat™ in Group 1 (*p* > 0.05). Similarly, in Group 2, factors such as gender, age, dfs score, GI, and PI were also found to have no significant effect on the overall clinical success of teeth treated with Fissurit FX (*p* > 0.05) (Table 5).

**Table 5:** Risk factors affecting the failure of pit and fissure sealants in Group 1 and Group 2

Groups	Risk Factors	Overall Clinical Success		Univariate	
		Successful	Unsuccessful	OR (%95 CI)	*p*
Group 1	Sex				
	Girl	29.00 (55.80)	3.00 (50.00)	Reference	
	Boy	23.00 (44.20)	3.00 (50.00)	1.08 (0.21–5.38)	0.919
	dfs score	7.62 ± 6.08	10.17 ± 7.25	1.05 (0.94–1.16)	0.369
	GI	0.67 ± 0.37	0.58 ± 0.39	0.51 (0.44–6.01)	0.597
	PI	1.20 ± 0.53	0.88 ± 0.56	0.34 (0.06–1.90)	0.062
Group 2	Sex				
	Girl	29.00 (55.80)	3.00 (50.00)	Reference	
	Boy	23.00 (44.20)	3.00 (50.00)	1.08 (0.21–5.38)	0.919
	Age				
	6.5–7.5	22.00 (42.30)	3.00 (50.00)	Reference	
	7.5–8.5	14.00 (26.90)	1.00 (16.70)	0.54 (0.05–5.25)	0.6
	8.5–9.5	16.00 (30.80)	2.00 (33.30)	0.78 (0.13–4.68)	0.788
	dfs score	7.62 ± 6.08	10.17 ± 7.25	1.05 (0.94–1.16)	0.369
	GI	0.67 ± 0.37	0.58 ± 0.39	0.51 (0.44–6.01)	0.597
	PI	1.20 ± 0.53	0.88 ± 0.56	0.34 (0.06–1.90)	0.062

GI, Gingival Index; PI, Plaque index; OR (%95 CI), Odds ratio (%95 Confidence Interval); Group 1, BioCoat™; Group 2, Fissurit FX

The number of evaluated teeth decreased over successive follow-up visits due to two reasons: (1) teeth that received a Charlie score at a given follow-up were considered clinical failures and were excluded from subsequent evaluations and (2) teeth belonging to patients who did not attend follow-up visits were excluded from further analysis. Therefore, the denominators vary across timepoints and are explicitly reported for each evaluation period.

## Discussion

Occlusal surfaces account for approximately 85% of dental caries in children, even though they represent only 12.5% of the total tooth surface area.This disproportion underscores the high susceptibility of pit and fissure regions due to their morphological characteristics [18]. PFS are recognized as an effective preventive approach for protecting these high-risk areas. However, reported failure rates of 5–10% within the first 12 months indicate that clinical success is highly dependent on both the application protocol and the material properties [19]. Recent breakthroughs in material science have led to the development of bioactive substances that elicit beneficial biological outcomes, including tissue repair, remineralization and antimicrobial action [20]. Recently, resin-based sealants with bioactive properties have been introduced, aiming not only to enhance mechanical retention but also to reduce the risk of secondary caries through ion release. Within this context, the present study was designed to compare the clinical performance of BioCoat™ and Fissurit FX over an 18-month period using a split-mouth design, thereby minimizing interindividual variability while evaluating intermaterial differences. This study represents one of the first studies to evaluate the long-term clinical outcomes of BioCoat™ and filling an important gap in the literature regarding the comparative performance of bioactive and conventional resin-based sealants.

In the present study, BioCoat™ and Fissurit FX demonstrated comparable clinical performance across all evaluated parameters. Although no statistically significant differences were observed between BioCoat™ and Fissurit FX in terms of marginal discoloration, numerical differences were noted. These differences did not reach statistical significance and, therefore, cannot be interpreted as evidence of superior performance. Similar findings have been reported in a comparative study evaluating BioCoat™ and Clinpro™, in which no significant differences were observed between bioactive and conventional resin-based sealants in terms of marginal discoloration, despite comparable clinical outcomes [16]. While previous studies have suggested that bioactive resin materials may contribute to improved marginal integrity by reducing microleakage [13, 14], the findings of the present study do not allow definitive conclusions regarding such material-related effects. In addition, the opaque white shade of Fissurit FX may have increased the visual detectability of marginal discoloration, which should be taken into consideration when interpreting these results.

In terms of marginal adaptation, both materials demonstrated comparable clinical performance. Although a higher frequency of adaptation loss was numerically observed in the BioCoat™ group, this difference did not reach statistical significance and should, therefore, be interpreted with caution. Consequently, these findings cannot be considered indicative of a clinically meaningful difference or superior performance of either material. Although it has been suggested in the literature that BioCoat's viscosity may potentially affect marginal adaptation [14], the results of the present study do not allow definitive conclusions regarding such effects.

In terms of retention, both materials demonstrated similar durability over an 18-month period. The presence of Alpha scores exceeding 70% further reflects this clinical stability. This similarity is likely explained by their common resin-based composition, the close similarity in filler loading percentages (BioCoat™: 56%; Fissurit FX: 55%), and the consistently applied standard application protocol across all cases [16].

With regard to secondary caries formation, no statistically significant differences were detected between BioCoat™ and Fissurit FX throughout the follow-up period. Although a higher number of Charlie scores were numerically observed in the Fissurit FX group at the 12- and 18-month evaluations, these differences did not reach statistical significance and, therefore, indicate comparable clinical performance rather than a definitive advantage of either material. Previous studies have suggested that bioactive resin materials may possess properties that could influence caries-related outcomes through ion release and remineralization processes [6, 10, 14]. However, independent clinical evidence supporting these mechanisms remains limited. To fully elucidate and realize the potential of bioactive materials with a focus on bioactivity, regeneration, and long-term oral health, sustained research and development efforts are required [20]. Accordingly, the findings of the present study do not allow definitive conclusions regarding material-related biological effects, and any interpretation of potential mechanisms should be made cautiously and requires further independent investigation.

After 18 months of follow-up, the findings supported the acceptance of the null hypothesis, showing that there was no statistically significant difference in overall clinical success between BioCoat™ and Fissurit FX. The success rate was 90.2% in the BioCoat™ group and 88.2% in the Fissurit FX group, demonstrating that both materials provide comparable efficacy in long-term clinical applications. Importantly, the success rates observed for Fissurit FX are consistent with previously reported findings [21, 22], strengthening the external validity of the current study. The structural similarities between the two materials (both are resin-based, fluoride-releasing materials with similar filler content) and adherence to standard clinical protocols (acid etching and rubber dam isolation) likely significantly contributed to these comparable clinical results. In addition, controlled variables such as preoperative plaque control, meticulous surface preparation, and operator standardization may have further supported the favorable long-term clinical outcomes observed in both groups [23].

Although both materials initially demonstrated 100% clinical success, a gradual decline in success rates was observed over time. At the 18th month follow-up, statistically significant reductions were identified in both groups compared with the 3rd and 6th month evaluations, suggesting that PFS may gradually lose mechanical and chemical resistance [12]. This observation is consistent with previous reports showing high early success rates that decline as the follow-up period extends [21, 24]. These findings indicate that the clinical performance of PFS is influenced not only by the quality of the initial application but also by the long-term durability of the material [22]. Consequently, incorporating survival analyses into material selection may provide more accurate and predictable clinical decision-making for long-term applications [25].

In the survival analysis, the median survival time was 17.72 months for BioCoat™ and 17.49 months for Fissurit FX. Although the median survival time was slightly longer in the BioCoat™ group, this difference was not statistically significant. Therefore, the survival outcomes indicate comparable durability between the two materials, and the observed numerical differences should be interpreted with caution. Similar findings have been reported in a 12-month clinical follow-up study comparing BioCoat™ and Clinpro™, in which no significant differences in survival outcomes were observed between fluoride-releasing resin-based sealants [16]. While previous in vitro studies have suggested that certain material formulations may influence resistance to occlusal stresses [26], the results of the present clinical study do not allow definitive conclusions regarding such material-related effects.

In this study, patient-related variables that could potentially influence clinical success, including gender, age, dfs score, gingival index (GI), plaque index (PI), and caries risk group, were evaluated. However, the caries risk category could not be statistically analyzed due to limited sample sizes within its subcategories, and age was excluded from statistical analysis in the BioCoat™ group because of non-homogeneous distribution. Gender was not identified as a statistically significant factor associated with clinical failure, which is consistent with previous long-term clinical studies reporting no determinative effect of gender on the performance of resin-based materials [27]. Although higher failure rates were numerically observed in male participants, this finding did not reach statistical significance. Previous studies have suggested that factors such as increased masticatory forces in males may influence mechanical fatigue of restorative materials [28], and that caries risk may increase with age due to dietary and hormonal factors [29]; however, the present study does not provide sufficient evidence to confirm these associations. Overall, these findings suggest that patient-related factors had a limited impact on clinical outcomes within the conditions of this study, while the split-mouth design may have contributed to minimizing interindividual variability and enabling a balanced comparison between the materials.

Regarding dfs scores, an upward but non-significant trend was observed, indicating that higher scores may be associated with increased failure rates. Similarly, although no statistically significant associations were detected between GI or PI indices and clinical failure, these indices may serve as indirect indicators of oral hygiene and biofilm accumulation. Collectively, these findings suggest that patient-related factors had only a limited impact on clinical success, whereas material properties appeared to play a more decisive role. The split-mouth design employed in this study may have further minimized interindividual variability, thereby reducing the potential influence of patient-related factors [30].

This study has several limitations that should be considered when interpreting the results. First, the single-center design may partially limit the generalizability of the findings. In addition, age and caries risk category could not be statistically analyzed in one of the study groups due to non-homogeneous age distribution and limited sample sizes within the caries risk subcategories. Consequently, the potential influence of these patient-related variables on clinical outcomes could not be fully assessed, which may restrict the generalizability of the results to broader pediatric populations with different age distributions and caries risk profiles. Another limitation is the loss of a proportion of participants during follow-up, which may have influenced the internal validity of the study, particularly with respect to survival analysis outcomes, despite the absence of evident baseline differences between retained and lost participants. Furthermore, the absence of operator and evaluator blinding, which was not possible due to visible color differences between the materials. Since all clinical procedures and outcome assessments were performed by the same experienced clinician, this may have introduced a risk of performance and detection bias. In addition, the opaque white shade of Fissurit FX may have increased the visual detectability of marginal discoloration, potentially influencing the evaluation of this outcome. These limitations should be taken into account when interpreting the findings.

A strength of the study is that the split-mouth design has provided a significant advantage by controlling inter-individual variability and enabling a direct comparison of material performance. Another advantage of this study is the clinical comparison of BioCoat™ with a conventional resin-based sealant over an 18-month follow-up, contributing to the limited body of long-term, controlled clinical research in this field. In addition, the use of modified USPHS criteria ensured that the outcomes were clinically meaningful and comparable. In this regard, this study provides a current and original data set that reflects real-world clinical performance rather than relying exclusively on laboratory-based outcomes.

Future studies with larger, multicenter samples and longer follow-up periods across acrosss age different age groups may provide stronger evidence on the clinical stability of bioactive PFS, their efficacy in preventing secondary caries, and their relationship with patient-specific variables. Furthermore, investigating alternative surface preparation protocols and material combinations could further enrich clinical decision-making process.

This study demonstrated that BioCoat™ and Fissurit FX showed comparable clinical performance over the 18-month follow-up period. Both materials performed similarly in terms of retention, marginal integrity, marginal discoloration, and secondary caries outcomes. Although bioactive properties have been proposed to offer preventive potential, the findings of the present study do not indicate a clinically relevant difference between the materials within the observed follow-up period. Within the limitations of the study, both BioCoat™ and Fissurit FX can be considered reliable and clinically effective options for pit and fissure sealant applications in paediatric dentistry.

## Trial registration

The study protocol was registered in the clinical trials database (www.clinicaltrials.gov) under the identifier NCT06987955.

## Data Availability

The datasets generated and/or analyzed during the current study are not publicly available due to ethical and privacy restrictions involving pediatric participants, but are available from the corresponding author on reasonable request.
